# From Shelf to Shelf: Assessing Historical and Contemporary Genetic Differentiation and Connectivity across the Gulf of Mexico in Gag, *Mycteroperca microlepis*

**DOI:** 10.1371/journal.pone.0120676

**Published:** 2015-04-09

**Authors:** Nathaniel K. Jue, Thierry Brulé, Felicia C. Coleman, Christopher C. Koenig

**Affiliations:** 1 Department of Biological Science, Florida State University, Tallahassee, FL, 32303, United States of America; 2 CINVESTAV-Mérida, Km. 6 Carretera Antigua a Progresso, A.P. 73, Cordemex, C.P. 97310, Mérida Yucatán, México; 3 Florida State University Coastal and Marine Laboratory, 3618 Coastal Highway 98, St. Teresa, FL, 32358, United States of America; 4 Florida State University Coastal and Marine Laboratory, 3618 Coastal Highway 98, St. Teresa, FL, 32358, United States of America; Chinese Academy of Sciences, CHINA

## Abstract

Describing patterns of connectivity among populations of species with widespread distributions is particularly important in understanding the ecology and evolution of marine species. In this study, we examined patterns of population differentiation, migration, and historical population dynamics using microsatellite and mitochondrial loci to test whether populations of the epinephelid fish, Gag, *Mycteroperca microlepis*, an important fishery species, are genetically connected across the Gulf of Mexico and if so, whether that connectivity is attributable to either contemporary or historical processes. Populations of Gag on the Campeche Bank and the West Florida Shelf show significant, but low magnitude, differentiation. Time since divergence/expansion estimates associated with historical population dynamics indicate that any population or spatial expansions indicated by population genetics would have likely occurred in the late Pleistocene. Using coalescent-based approaches, we find that the best model for explaining observed spatial patterns of contemporary genetic variation is one of asymmetric gene flow, with movement from Campeche Bank to the West Florida Shelf. Both estimated migration rates and ecological data support the hypothesis that Gag populations throughout the Gulf of Mexico are connected via present day larval dispersal. Demonstrating this greatly expanded scale of connectivity for Gag highlights the influence of “ghost” populations (*sensu* Beerli) on genetic patterns and presents a critical consideration for both fisheries management and conservation of this and other species with similar genetic patterns.

## Introduction

Understanding the genetic structure of populations requires distinguishing the signature of historical influence from the consequences of contemporary patterns of migration and population size [1, 2]. Describing population structure and delineating its causes is not only necessary for understanding the basic process of species and population evolution, but it often guides conservation and management decisions [3]. Many studies of genetic population structure have uncovered the role of historical barriers to dispersal, which created congruent patterns of highly subdivided populations [4]. A prominent example in maritime phylogeography is the role of the Florida peninsula as a principal geographic barrier creating patterns of differentiation among populations of various species [5–7]. However, focusing on genetic differentiation across these types of specific barriers can sometimes obscure phylogeographic patterns [8].

Wide-ranging taxa that display little or no spatial differentiation in genetic variation present a different interpretive challenge. Many reef-associated marine species found in the southeastern United States (including in the Gulf of Mexico (GOM) and along the Atlantic coast) show this particular pattern of having an extended range with low levels of present day population differentiation [9–15]. In these cases, the challenge involves distinguishing a temporally shallow history of rapid range expansion from a long history of established populations with regular exchanges of migrants [16, 17]. Additionally, excluding populations that may contribute to gene flow can result to misleading interpretations of observations [18, 19]. For instance, although many of those aforementioned Gulf and Atlantic reef species are also found along the Yucatan Peninsula on Campeche Bank (CB), little is known about the relationships of these populations to others in the southeastern United States. Thus, our perspective on the evolutionary and ecological genetics of the region is limited.

Developments in population genetic methods [20–22] have highlighted the importance of both historical and contemporary processes and our ability to pre-suppose factors driving population genetics. For example, estimates of migration rates and time of divergences in big-eye tuna (*Thunnus obesus)* revealed sizeable and asymmetrical migration patterns between Atlantic and Pacific populations, inconsistent with population genetics determined solely by historical factors [23], while, conversely, a similar approach showed two newt subspecies to be maintaining historical genetic differentiation despite recent gene flow [24]. Even more complicated comparisons such as the secondary contact described in Duvernell et al’s [25] assessment of mutation-drift equilibrium and patterns of genetic structure and gene flow in the Mummichog, *Fundulus heteroclitus*, reveal the complexity that the intersection of historical and contemporary processes can generate. In each of these cases, we have gained a greater understanding of factors affecting population genetics and their relationship to understanding current species biology.

Gag, *Mycteroperca microlepis*, an epinephelid fish common in the Gulf of Mexico and Western Atlantic and target for important regional fisheries, illustrates these issues related to the population genetics of wide-ranging marine species. Gag possess a complex life cycle and a set of distinct life history that are broadly representative of many reef fishes in this region. This includes ontogenetic migrations among diverse habitats over the course of their life time, from an open ocean pelagic larval stage lasting about 40 days, to seagrass associated juvenile stage for 7–9 months [26–28], and an adult stage largely confined to offshore shelf-edge habitats (40–60 m depths). They form relatively large (~100 individual) aggregations to spawn from late winter to early-spring and have a subsequent larval period of ~40 days, after which they move into nearshore seagrass beds [26–28]. In autumn, juveniles migrate from seagrass beds to nearshore patch reefs. As they approach sexual maturity (around ages 3–5), they move to offshore patch reefs and the high relief, continental shelf edge spawning habitats, with females moving on and off aggregation sites seasonally while males remain on these sites year round [29]. Individuals spawn initially as females, with some small portion of the population transforming to males as they approach 10 years of age. Despite this ontogenetic dependency on regionally and locally discontinuously-distributed habitats, Gag show little genetic structure among widely separated adult populations [12] or among juvenile cohorts in seagrass beds [30, 31].

Studies on other species from the GOM provide little insight as to whether this lack of spatial structure in Gag is because of historical or contemporary processes. Fluctuation in sea level and large oscillations in the size of the GOM over evolutionary time have been suggested as mechanisms for homogenizing genetic variation among different reef fish populations, while also explaining why population genetics reflect recently expanded rather than historically isolated populations [32, 33]. Alternatively, continued migrant exchange could homogenize contemporary genetic structure. Although unlikely given the distances and lack of intervening adult habitats, tagging studies have shown that long-distance dispersal due to adult migration occurs in Gag, albeit at low numbers [34, 35]. Ecological evidence combined with dominant oceanographic current structure suggest that long-distance larval dispersal between the Mexico and West Florida continental shelves across the Yucatan Channel (2800 m deep) is possible [36, 37]. Evidence of historical and/or contemporary connectivity between the two regions would offer considerable insight into regional phylogeographic patterns and the contemporary genetic relationships among populations, while informing on-going conservation and management efforts of this and other important fishery species.

In this study, we examined genetic variation in Gag populations on Campeche Bank and the West Florida Shelf to evaluate hypotheses about emerged patterns that appear to be explained by contemporary gene flow. By using a variety of genetic data and implementing traditional and coalescent-based methods, we distinguish the signature of history from the pattern produced by ongoing migration, providing evidence for regional population connectivity previously underappreciated in contemporary population genetic assessments.

## Methods

### Sampling

Genetic samples (fin clips and heart tissue) for adult Gag were collected in conjunction with collaborative research efforts with commercial fishermen along the West Florida Shelf (WFS) [29] and fisheries-dependent port sampling on CB along the Yucatan peninsula. Fish capture and sample collection followed established animal care protocols approved by the Institutional Animal Care and Use Committee of Florida State University (Protocol Number: 9902). All sampling in United States waters occurred under Federal Permit F SER24:PH and State of Florida Permit SAL-12-1244-SRP. All Mexico samples taken post-mortem from commercial fishermen, and, thus, were exempt from requiring a sampling permit. Samples were stored in a Sarcosyl-Urea solution (1% n-lauryl sarcosine, 8 M urea, 20 mM sodium phosphate, and 1 mM EDTA, pH 6.8) at room temperature.

### Molecular methods

Genomic DNA was extracted using magnetic beads methodology (Agencourt, Inc., Beverly, MA, U.S.A.). Microsatellite primers for 10 loci, developed in other studies for Gag [31], black grouper, *Mycteroperca bonaci* [38], red grouper, *Epinephelus morio* [11], and Hawaiian grouper *Epinephelus quernus* [39], were assayed using fluorescently labeled primers (IDTDNA, Applied Biosystems, Foster City, CA, U.S.A.). All samples then underwent multiplexed polymerase chain reaction (PCR) amplification and subsequent genotyping at these 10 loci. Standard PCR conditions were used for all reactions. Any loci that exhibited ambiguities were run independently for validation. PCR protocols varied among loci and are available from the author upon request. Samples were analyzed on an Applied Biosystems (ABI) 3130xl Genetic Analyzer with Capillary Electrophoresis. Data were read and analyzed using ABI Genemapper Software version 4.0.

A 500–900 base pair segment of the Gag mitochondrial control region was sequenced using a set of universal primers: L15926 [40] and H16498 [41]. These primers are in codons for evolutionarily conserved amino acids flanking the control region and have previously been used for several species of fishes, specifically cichlids (Meyer *et al*., 1990). The high-fidelity Invitrogen Pfx50 Taq was used to minimize sequencing errors. PCR products were direct sequenced using PCR primers. All samples were sequenced with both forward and reverse primers and no sequence was included in the data set where contigs of those two sequences could have yielded ambiguous results (any repetitive regions required both flanking regions to be sequenced appropriately). All sequence ambiguities were examined, edited manually, and aligned using Sequencher v.4.5 software. Two perfect-repeat indels were found in this region (S1 Fig). One is a 9-bp repeat (CATTAATTA) and the other is a 40-bp repeat (TCTGTACAATGGTTCAAATACGCAATATGTTCCATCATCA). Repeats were analyzed both separately as RFLP haplotypes and concurrently as a scored character state for presence/absence. Data were combined into one data set by converting all indels into single nucleotide site character. For example, a sample with two 9-bp and seven 40-bp repeats would have two and seven adenosines replace those repeats in sequence data, respectively, and single site gaps for every missing repeat. For the 9-bp and 40-bp repeats, each motif repeated a maximum of seven and thirteen times, respectively.

The haplotype sequences generated and used in this work were deposited in the GenBank (accession numbers KM888545-KM888674). All raw genotypic and sequence data can be found in S1 and S2 Files.

### Population structure

**Fig 1:**
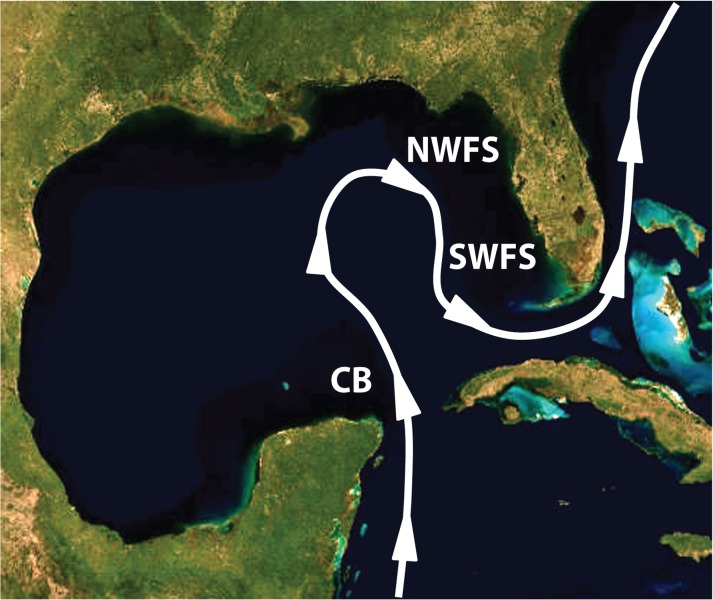
Map of the Gulf of Mexico showing Campeche Bank (CB) and the two regions of interest on the West Florida Shelf (WFS): the North West Florida Shelf (NWFS) and the South West Florida Shelf (SWFS). Solid line and arrow indicate general location and direction of the Gulf Loop Current and Gulf Stream Current in the region. Image was taken from U.S. Geological Survey, National Geospatial Program website.

Patterns of genetic differentiation across the GOM were examined using both traditional F-statistic-based [42] and coalescent-based approaches (see below). The grouping schemes in this study were used to address different hypotheses of “population” delineations based on existing biological evidence and the degree of genetic support for them. The first scheme contained two “populations” representing Campeche Bank and the West Florida Shelf to assess broad-scale regional effects. The second scheme, delineated samples into three “populations” consisting of Campeche Bank, the West Florida Shelf north of latitude 28° N (NWFS) and the West Florida Shelf south of latitude 28° N (SWFS) (Fig 1) to provide a more in-depth comparison of regions assumed, *a priori*, to be experiencing high migration rates (those in close proximity to one another on the WFS) and regions that may be experiencing much lower migration rates (Campeche Bank and the West Florida shelf). The rationale for the second scheme is based on previous studies that demonstrated (1) spatial differences in growth patterns of a related species, the Red Grouper *Epinephelus morio* along the WFS, suggesting possible population structure [43]; (2) spatial differences in arrival times of juvenile Gag recruits [36], and (3) genetic evidence from other work demonstrating weak spatio-temporal differentiation among these areas [44]. Loci were examined to assess adherence to underlying assumptions about neutrality and independence. Microsatellite loci were tested for significant linkage disequilibrium and deviations from Hardy-Weinberg equilibrium using the program Genepop [45] as well as null alleles and allelic drop out using the program Microchecker [46]. Control Region sequences and indel regions were tested for neutrality using Tajima’s D [47] and Fu’s Fs [48]. Calculations were conducted in Arlequin v.3.1.1 [49], with mtDNA being broken up into three different test groups: sequence only, sequence + indels, and indels as RFLPs. Additionally, measures of genetic diversity and other marker specific summary statistics were calculated.

Genetic differentiation among comparison groups was assessed across all loci. Microsatellite data were tested for differentiation among “populations” using Chi-squared tests [50] and Fisher’s Exact Tests [51] using the program CHIFISH [52]. Exact Tests probabilities were calculated using a Markov Chain approach that employed 10,000 dememorization steps, 100 batches, and 5000 iterations of each batch. Power and rate of false positives for regional and sub-regional comparisons were conducted for these tests using the program POWSIM v.4.0 [53]. Mitochondrial loci were tested for differentiation using Fisher’s Exact Test. Unrooted minimum spanning networks based on sequence only and sequence and indel information were drawn from distance matrices generated in Arlequin v.3.1.1 in which each step represented either a base change or indel number change.

### Historical population dynamics

We explored the role of historical events on the present distribution of genetic diversity in several ways. Patterns in microsatellite gene diversity relative to equilibrium expectation were assessed using the program Bottleneck v.1.2.02 [54], implementing a Two-Phase mutational model (TPM). Deviation from equilibrium expectations indicates either heterozygosity excesses due to population bottlenecks or deficiencies due to population expansion and/or migration. Support for models of historical demographic and spatial expansion [55, 56] was examined using mismatch distributions of mtDNA control regions [57, 58] using parameter bootstrapping. Mismatch distributions use patterns of pairwise differences among haplotypes to estimate time since population expansion and assess whether distributions of differences among sequences match respective model expectations. These models predict patterns of generic variation based on population growth or range expansion. Raggedness indices were also calculated to evaluate evidence for multi-modal distributions indicative of stationary populations [59]. Sequence and repeat RFLP data were examined separately because of their different mutational nature. The time of expansion, τ, was estimated along with the mutation parameters theta initial, θ0, and theta final, θ1. Actual dates of expansion were estimated using the equation τ = 2μtG, where μ is the mutation rate, t is the number generations since expansion, and G is generation time (7.94 yrs. for an unfished population; N. Jue, unpublished data). The mutation rate of fish mitochondrial control region was generated by averaging estimates from 8 other species (μ = 0.02888 changes/site/Myr) [60]. Subsequent mutation rates for sequence only, indel repeats, and combined data were 1.23 x 10–4, 3.67 x 10–6, and 1.26 x 10–^4^, respectively.

### Migration patterns

Patterns of genetic connectivity for Gag in the GOM were examined using Bayesian coalescent approaches [22, 61]. Migration patterns were principally estimated from microsatellite data using the coalescent-based program MIGRATE v3.1.2 [21]. Exhaustive attempts were made to do the same with mitochondrial data, but Markov chain Monte Carlo (MCMC) runs would not converge to a reliable result. Estimation of parameters in MIGRATE was done using the following Bayesian approach that proved to be the most effective in identifying the true patterns of migration [61]. Analyses for all cases consisted of running 50 replicate chains of 500 recorded steps with a 100-step increment between recorded steps on a data set consisting of all available samples from CB (n = 85) and an equal number of samples (n = 85) randomly chosen without replacement for both NWFS and SWFS. Samples from NWFS and SWFS were lumped for the 2-population case runs. A 1,000,000 step burn-in was applied to these runs as well as an 8-chain thermodynamic static heating scheme (temperatures of 1,000,000.00, 7.00, 3.50, 2.33, 1.75, 1.40, 1.17, and 1.00); thermodynamic static heating schemes have been shown to yield the best estimates of marginal likelihoods [61] and the heating scheme chosen represents a moderately heated scheme. Relative mutation rates were estimated from the data and posteriors were sampled using a Slice sampler. A uniform prior with the interval [0, 100] and a delta-value of 10 was used for estimating mutation-scaled effective population size θ (= 4N_e_μ) and mutation-scaled migration rates M (= m/μ) among groups. Finally, number of migrants (Nm) was calculated by using the generated estimates for θ and M (Nm = (θ*M)/4).

MIGRATE can accurately differentiate among migration models to find the one that is best supported by the data [62]. It provides marginal likelihoods for specific migration models using chain heating and thermodynamic integration. These scores can be implemented in Bayes Factor comparisons [63] and, given large differences in fit (Bayes Factor ratios >150:1), used to describe significant support for one model over another. In this study, different migration models were compared to each other to test which hypotheses best explained patterns of genetic variation in Gag. As with patterns in population differentiation, the various models tested in this study reflect our interests in both the grouping of “populations” and the degree and nature of connectivity among them. Based on previously mentioned scheme for examining population differentiation, the same samples were divided into 1-, 2-, or 3-sub-population model, with the 1-population model representing panmixia, the 2-population models representing the CB or WFS regions (1^st^ scheme), and the 3-population model breaking up the WFS into NWFS and SWFS samples (2^nd^ scheme).

While population size varied freely, migration type and directionality was strictly defined for various hypothetical “scenarios” (i.e. models) in order to examine hypotheses about what types of connectivity best describes the data. These migration “scenarios” included default models of migration among populations allowing for either unfettered asymmetric or symmetric migration among all populations (a symmetric migration model is analogous to the Island-Model assumptions associated with traditional F_ST_ analyses) and specific models based on other regional Gag migration studies. These specific cases included the following 3-population examples: *Scenario 1*: CB as a genetic source population and WFS sub-populations as sinks (i.e. one-way migration from CB to both WFS populations and free migration within the WFS); *Scenario 2*: a migration model allowing connectivity among all population except for no migration from SWFS to CB; and *Scenario 3*: WFS as genetic source populations and CB as a sink (i.e. one-way migration from the WFS to CB and free migration within the WFS). *Scenario 1* summarizes a connectivity hypothesis describing either historical dispersal from CB or on-going connectivity via larval dispersal [36]. *Scenario 2* reflects the combination of ecological hypotheses on connectivity from various ecological studies of Gag dispersal in the GOM [34–36]. Given the barriers to contemporary larval dispersal from WFS to CB described above, *Scenario 3* models either historical secondary contact due to events such as historical range contractions typical to glaciation events or on-going adult migration from the WFS around the GOM to CB. Bayes Factors were calculated for all comparisons and values greater than 150 are considered very strong support for a particular model.

In addition to MIGRATE, the coalescent-based program IMa [22] was used to further assess the roles of population growth and migration by simultaneously estimating migration rates between CB and WFS, population sizes for CB, WFS, and their ancestral population, and time since divergence. Two replicate runs of IMa were run with all data microsatellite and mtDNA combined (mtDNA was broken up into two loci, one with sequence information and another with repeat information on number of indels). One additional run using just microsatellite data was made to assess effect of mtDNA on overall pattern. Metropolis coupling runs were also used; these implement 75 multi-Markov coupling chains and a geometric increment model heating scheme, which consisted of a 500,000 step burn-in period followed by a time dependent run that lasted over 700 hours. Each run covered about 4,000,000 steps with 40 steps between samples. Scalars for population size and migration were set at 10 and the maximum time of splitting was set at 5. Run convergence was assessed by looking at the repeatability of result, comparisons between the first half and second half of the run, posterior distributions of parameter estimates, estimates of effective sample sizes (ESS), and the rate of swapping among successive chains. Averages of the parameter-value bin with the highest residence time from the 2 runs consisting of all the data were reported as the best parameter estimate.

## Results

A total of 474 fish were used in this study, 85 from CB, 148 from SWFS, and 241 from NWFS. All fish were genotyped for microsatellite loci with < 0.4% of the data missing (S1 File). The number of alleles per locus ranged between 4 and 33 (S1 Table). Loci across all populations showed high heterozygosities and limited variation among populations in allele richness (S1 Table). Of the 474 samples, mitochondrial control region sequences were generated for 47, 39, and 44 individuals from CB, SWFS, and NWFS, respectively (S2 File). Summary statistics of mtDNA data varied depending upon how the data were examined (S2 Table). Sequence data showed NWFS to have the largest value for θ, followed by CB and then SWFS; while repeat RFLP data showed CB to be the largest, followed by SWFS, and then NWFS. Combined data summaries reflected the RFLP data patterns.

The data were largely consistent with neutral expectations. Tests for Hardy-Weinberg Equilibrium (HWE) showed little evidence for significant deviations from equilibrium expectations for microsatellites. Only three tests of the ten loci within population supported significant deviations after Bonferroni correction, but no consistent pattern of bias could be determined for any one locus across all populations. No significant evidence for null alleles or large allele drop-out was observed. Loci exhibited variable levels of linkage disequilibrium among populations (S3 Table); however, no instances were significant after Bonferroni corrections. For mtDNA sequence, sequence-only data generally showed significantly less genetic variation than expected under mutation-drift equilibrium, while RFLP data showed no consistent pattern of deviation from neutral expectations (S2 Table).

**Fig 2:**
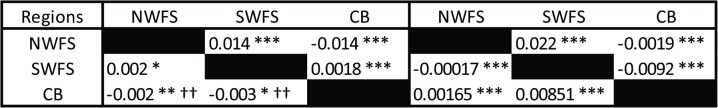
Pairwise measures of genetic differentiation among gag populations in the Gulf of Mexico. Left section of table shows standardized measures of genetic differentiation (φ’_ST_) of microsatellite markers below the diagonal and φ_ST_-values of all mtDNA data (sequence and indel repeats) above the diagonal. Right section of table shows φ_ST_-values for sequence data only and φ_ST_-values for mtDNA indel repeats above the diagonal. “*” denotes population significantly different using exact test; “†” denotes populations significantly different using χ2-test. Number of each respective symbol indicates level of significant (e.g. * is p < 0.5; ** and †† are p < 0.01; *** is p < 0.001).

We found statistically significant population structure across the GOM, but at a very low magnitude (Fig 2). Both NWFS and SWFS showed significant differentiation from CB across all data comparisons (nuclear and mtDNA for 2^nd^ scheme comparisons). While the NWFS and SWFS appeared to be significantly different from each other, those differences disappeared after the application of Bonferroni corrections. Two-“population” comparisons of WFS and CB (1^st^ scheme comparisons) also revealed significant differentiation using both Fisher’s Exact Test (p-value = 0.00024) and a Chi-squared Test (p-value = 0.00002). Tests possess sufficient power of detectability as POWSIM runs, using the allele frequencies determined from the empirical data, yielded false positive rates less than 5%. While we found some evidence of differentiation, the magnitude of this difference was quite small (all indices < 0.03). This includes standardized measures of microsatellite F_ST_-values (φ’_ST_), which compensate for high-heterozygosity bias, and, thus, low-magnitude values, associated with these loci. In fact, significant differentiation was often found where pairwise comparisons showed a negative value (effectively equal to zero) for φ’_ST_. These results indicate that while significant genetic differentiation may exist between regions (particularly between CB and WFS populations), the effect size of such differences was small.

**Fig 3:**
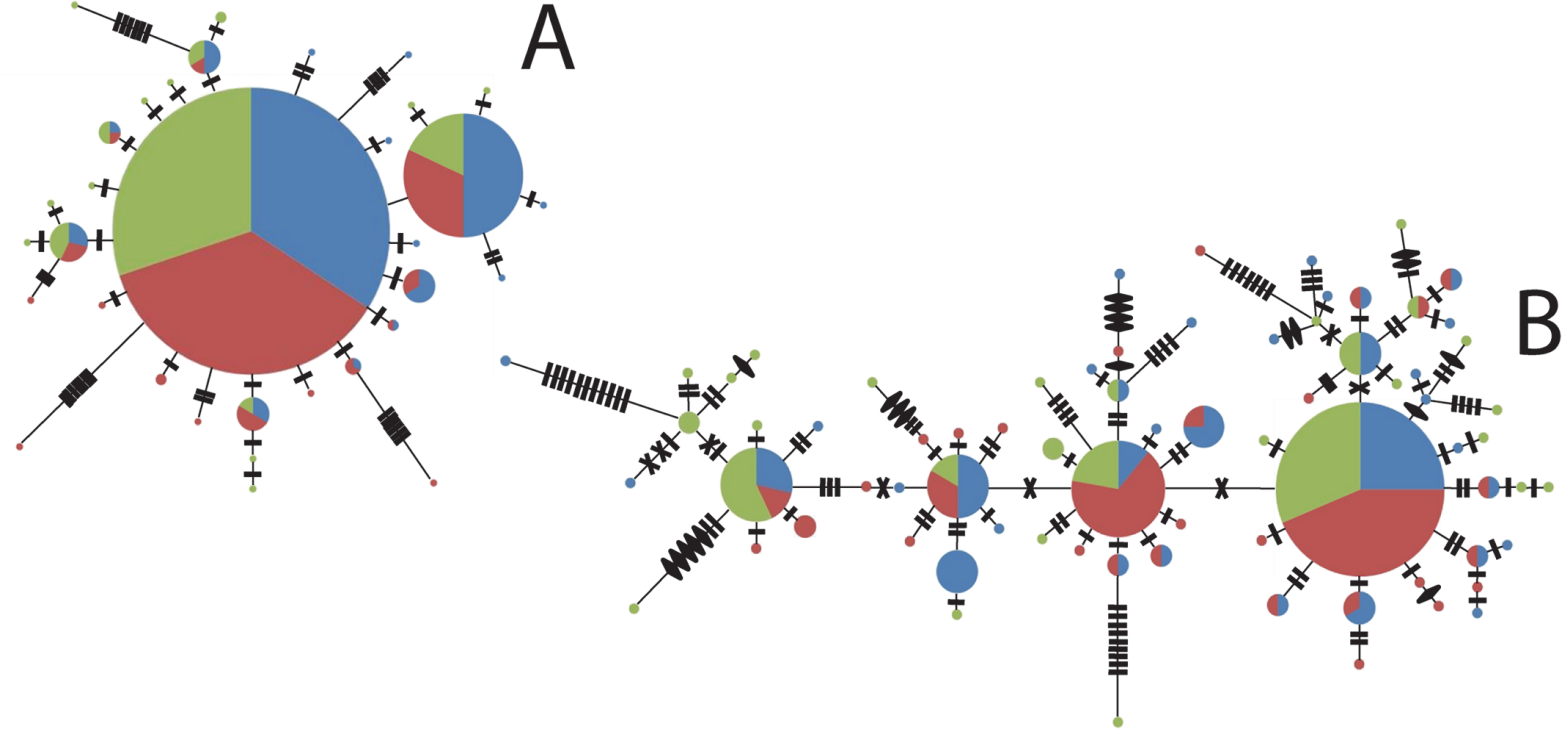
Unrooted minimum spanning network for mtDNA Control Region sequences. A represents sequence data only; B represents sequence and indel data. Each circle represents a haplotype where the size of the circle is directly proportional to haplotype frequency and pie slices indicate proportions of haplotypes from each region (Campeche Bank—black; South West Florida Shelf—dark grey; North West Florida Shelf—light gray). Hash marks (**█**) indicate 1 step difference between sequences. Crosses (**χ**) indicate a 40-bp indel repeat difference. Diamonds (♦) indicate a 9-bp indel repeat difference.

We found a similar low magnitude of population differentiation (Fig 2) in the mtDNA. Unrooted minimum spanning networks did not support the distinct spatial distributions of any specific haplotype lineages (Fig 3). Sequence-only haplotype networks revealed a star-like formation often associated with population events such as population expansion and combined data indicated an even more diverse set of haplotypes shared across all populations. In both cases, NWFS samples had lower levels of the haplotype diversity than those from other regions (see also S2 Table), although much more so when the RFLP data was included (Fig 3B) than when it was not (Fig 3A). Overall, there was little evidence for strong spatial distinctions and, thus, lineage sorting appears incomplete, indicative of a shallow population history or significant migration among populations.

**Fig 4:**
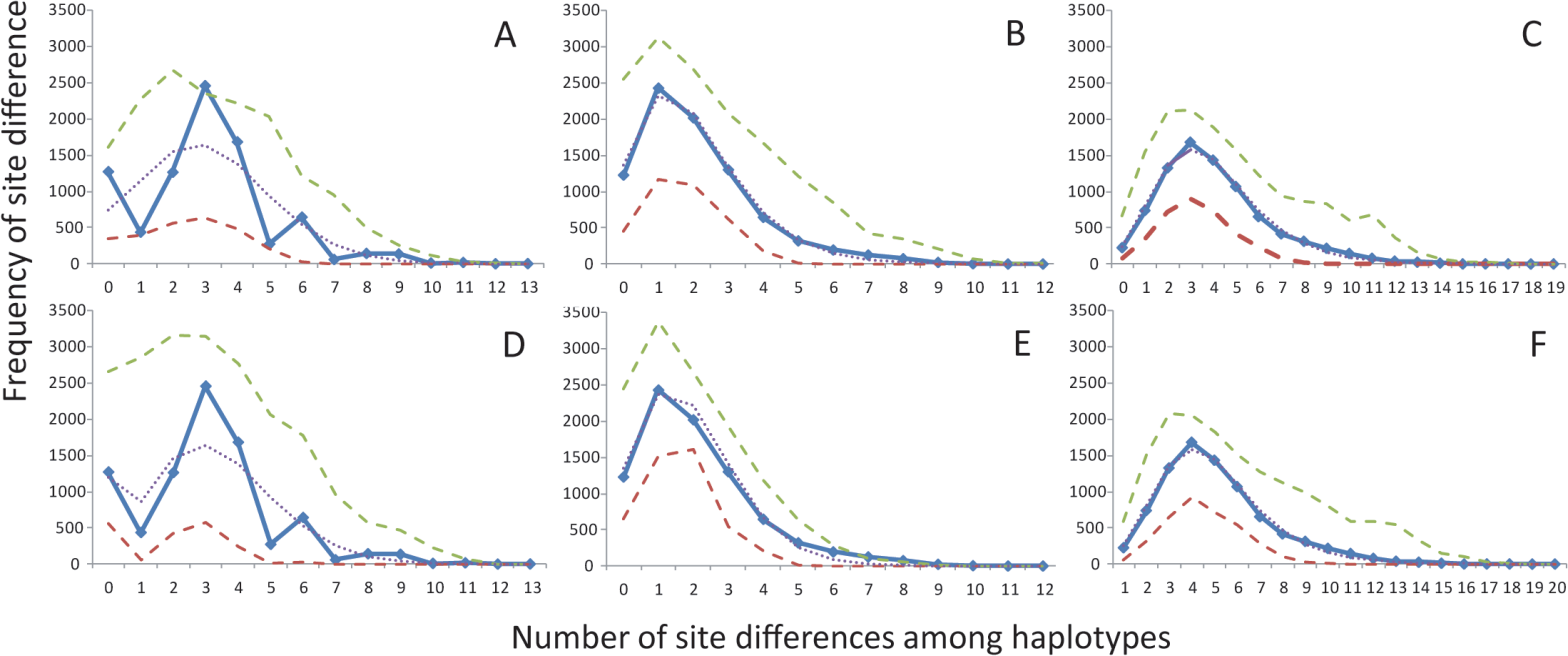
Mismatch distributions of mtDNA haplotypes from pooled Gulf of Mexico samples. *A-C* show results from fitting data to a pure demographic expansion model and *D-F* show results from fitting data to spatial expansion model. *A* and *D* are derived from sequence information only, *B* and *E* are derived from indel information only, and *C* and *F* are derived from both data-types combined. The demographic expansion model was only rejected for *A*. In all other case, demographic and spatial expansion models were not rejected. Solid line represents observed data; dotted line represents model prediction; dashed line represent 95% confidence interval for model prediction.

Genetic data suggested that patterns of genetic diversity were influenced by either demographic or spatial expansion. Mismatch distributions of mtDNA fit both pure demographic and spatial expansion models; however, different models were diagnosed as “best fit” when different subsets of the data were used. For sequence-only data, spatial expansion models could not be rejected from any level of examination (i.e. Fig 4D and 4F); however, demographic expansion models were a good fit for WFS populations only (Table 1). They were rejected for both the entire GOM and CB mismatch distributions (Fig 4A; Table 1). Additionally, significant raggedness was detected for these two sample groupings (Table 1), indicating population stability over long periods of time. Thus, spatial expansion within the GOM and from CB to WFS would appear to be the best fit to the data. Results from indels (Figs 4B and 3E) and combined data (Fig 4C and 4F) mismatch distributions fit equally well to both demographic and spatial population expansion and showed no evidence for significant raggedness. For microsatellite data, all permutations of Bottleneck analyses, except for NWFS, showed evidence of heterozygosity deficiencies (CB showed only marginal significance in this regard) (Table 2). Population bottlenecks typically leave a signature of heterozygosity excesses, whereas population expansion or undetected population substructure with gene flow lead to heterozygosity deficiencies. Thus, evidence suggests that either population expansion or substructure led to current genetic states as opposed to historical bottlenecks.

**Table 1:** Estimates of demographic and spatial expansion model parameters and raggedness indices based on mismatch distributions.

**Demographic Expansion**	**5% qt (τ)**	**E(τ)**	**95% qt (τ)**	**5% qt (θ**_0_ **)**	**E(θ**_0_ **)**	**95% qt (θ**_0_ **)**	**5% qt (θ**_1_ **)**	**E(θ**_1_ **)**	**95% qt (θ**_1_ **)**	**Model *p*-value**	**Raggedness (*r*) Index**	***r p*-value**
Gulf of Mexico	1.53	3.5	4.76	0	0	0.84	6.65	14.06	99999	0.05	0.084	0.03
- Campeche Bank	1	3.5	4.57	0	0.0018	1.42	5.82	11.94	99999	0.03	0.11	< 0.001
- West Florida Shelf	0.83	2.9	4.1	0	0.037	0.47	5.69	9.29	99999	0.53	0.029	0.73
—South West Florida Shelf	0.63	1.5	1.96	0	0.007	0.4	4.57	99999	99999	0.59	0.066	0.34
—North West Florida Shelf	0.91	2.5	4.13	0	0.019	0.11	6.13	9.53	99999	0.4	0.47	0.53
**Spatial Expansion**	**5% qt (τ)**	**E(τ)**	**95% qt (τ)**	**5% qt (θ**_**S**_ **)**	**E(θ**_**S**_ **)**	**95% qt (θ**_**S**_ **)**	**5% qt (M)**	**E(M)**	**95% qt (M)**	**Model *p*-value**	**Raggedness (*r*) Index**	***R* p-value**
Gulf of Mexico	0.74	3.4	4.8	0.0007	0.0007	1.95	2.88	7.92	99999	0.27	0.084	0.26
- Campeche Bank	1.33	3.3	5.087	0.0007	0.0007	1.36	3.25	7.013	161.18	0.16	0.11	0.19
- West Florida Shelf	1.17	2	3.62	0.0007	0.75	1.4	4.037	13.83	99999	0.56	0.29	0.64
—South West Florida Shelf	0.83	1.5	1.87	0.0007	0.0063	0.57	8.38	99999	99999	0.49	0.062	0.46
—North West Florida Shelf	0.54	2.4	3.39	0.0007	0.0007	1.53	2.56	11.62	99999	0.53	0.047	0.54

τ is the time of expansion. Θ_0_ is initial population size. Θ_1_ is final population size. Θ_s_ is the population size for spatial expansion. M is the estimate of number of effective migrants (2Nm, where N is the population size and m is the migration rate during expansion). Model p-value shows significant difference from model prediction if p < 0.05. Raggedness index summarizes multi-modality of mismatch distribution with r p-value < 0.05 indicating significant deviations from uni-modal distribution.

**Table 2:** Results from Bottleneck analysis of Gulf of Mexico microsatellite data.

	Two-phase Mutational Model				
	GOM	CB	WFS	SWFS	NWFS
Significant deviation	0.0098	0.10	0.0098	0.018	0.38
He excess	1.0	0.96	1.0	0.99	0.84
He deficiency	0.0049	0.052	0.0049	0.0093	0.19
Sign Test	0.014	0.066	0.0022	0.014	0.064

Data was divided up into different groups for analysis depending on the scale of interest. P-values for tests of deviations from expected heterozygosities for a two-phase mutational model are reported. The first three rows show results for Wilcoxon sign rank test with “Significant deviation” indicating a two-tailed test (row 1) and He excess(row 2) or deficiency (row 3) indicating a one-tailed test. The last row shows results from the less powerful Sign Test.

Time-since-expansion (τ) estimates indicated that any expansion predates the Holocene. All estimates were nested within the Pleistocene and were generally similar for both demographic and spatial expansion models. Times-since-expansion for spatial expansion models in the entire GOM were 109,740 (95% C.I. = (23,885; 154,927)) years ago (ya), 140,627 (95% C.I. = (76,804; 319,114)) ya, and 85,702 (95% C.I. = (63,016; 142,416)) ya for sequence, RFLP, and combined data, respectively. Interestingly, GOM and CB showed similar value for τ in both models for sequence data. WFS, SWFS, and NWFS all showed lower values for τ than CB (Table 1). Thus, while estimates of time since expansion in all populations are on similar time scales, those on the WFS appeared to be younger than those on CB.

**Fig 5:**
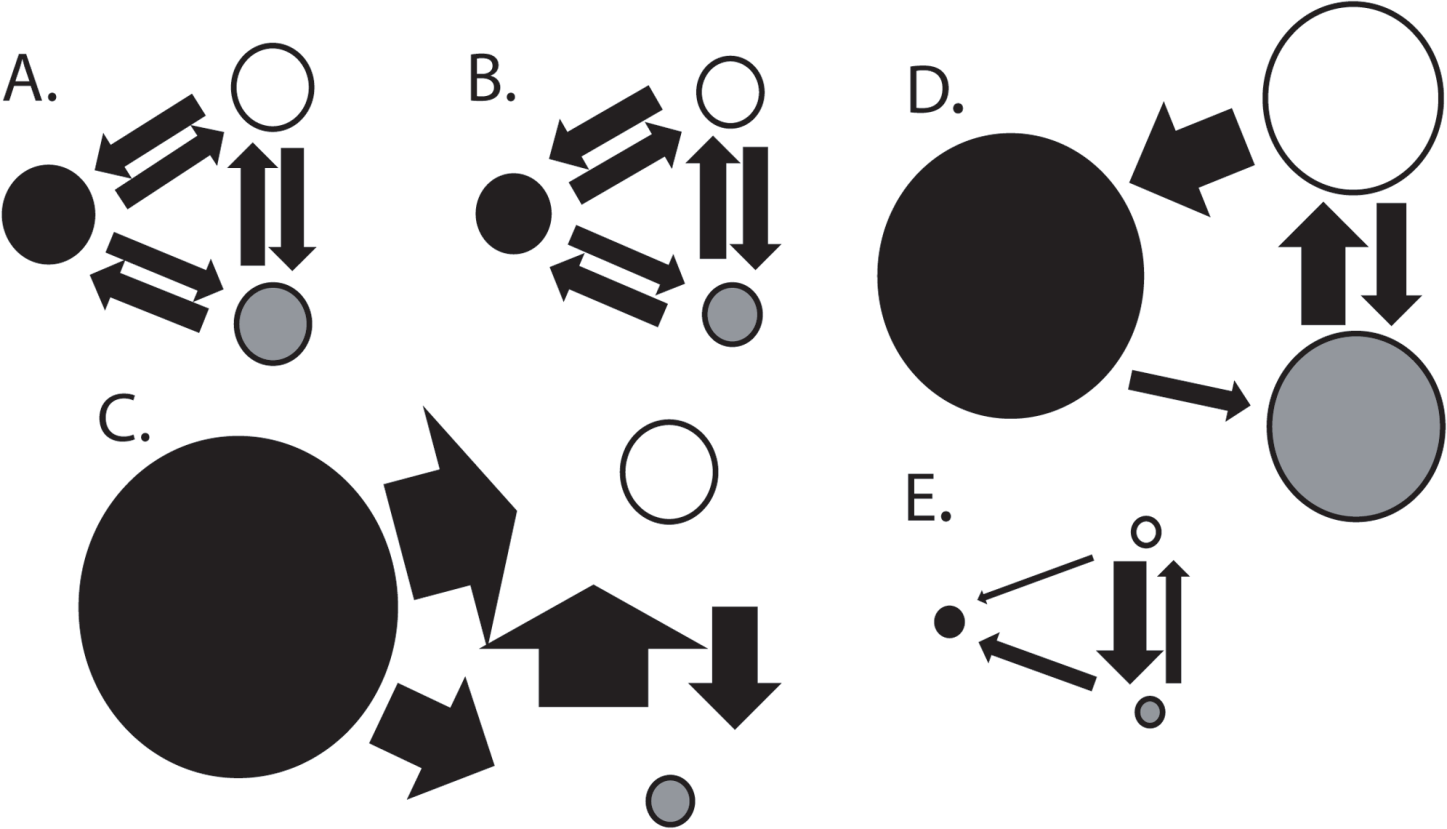
Schematics of three migration networks describing relative Θ and Nm-values. A—Full migration model; B—Symmetric migration model; C—*Scenario 1*: Campeche Bank as source population; D—*Scenario 2*: Connectivity matrix based on ecological data; E—*Scenario 3*: West Florida Shelf as source population. All circle and arrows sizes are, respectively, proportional to estimates of theta and number of migrants per generation (see S4 Table for values). Black circles represent Campeche Bank population; Grey circles represent South West Florida Shelf; White circles represent North West Florida Shelf. Bayes Factor model comparisons showed C to be very strongly supported (>> 150) by the data over all other migration models.

Runs of the program MIGRATE v3.1.2 on the microsatellite data described genetic migration patterns dominated by asymmetric gene flow. The ranking of migration models based on Bayes Factor (BF) scores resulted in the following hierarchy (best-fit model to worst-fit model; all comparisons showed very strong support for ranking difference (BF >150)): one-way migration from CB to NWFS and SWFS (*Scenario 1*), the 3-population migration model which mimicked available ecological data (*Scenario 2*), full 3-population migration model, full 3-population symmetric migration model, one-way migration from CB to WFS, one-way migration from WFS to CB, full 2-population model, full 2-population symmetric model, one-population model, one-way migration from NWFS and SWFS to CB (*Scenario 3*). Except the poorly fitted *Scenario 3* model, CB showed consistently higher estimates for θ than the other populations (Fig 5, S4 Table) in all 3-population model runs despite hypothesized smaller contemporary population size as compared to the WFS. This larger θ-value is congruent with the mitochondrial data which suggested CD to be an older population with persistent population stability. In the best supported model (*Scenario 1*), patterns of migration, based on the estimated number of migrants (Nm), showed substantial migration from CB to both NWFS and SWFS (Nm > 20) that was on the same scale as that between NWFS and SWFS (S4 Table). In the top five best supported models, all described migration from CB to the WFS with a migration rate ≥ ~5 Nm per generation, supporting the idea of significant ongoing connectivity with WFS populations resulting in low magnitude population differentiation. Runs of IMa supported these results, although they did not completely converge for one parameter. Of the six parameters of interest (θ_CB_, θ_WFS_, θ_ancestral_, m_CB->WFS_, m_WFS->CB_, τ), five converged on estimated values (θ_CB_ = 8.35; θ_ancestral_ = 14.58; m_CB->WFS_ = 1.14; m_WFS->CB_ = 0.18; τ = 0.908), while one (θ_WFS_ = 1837.06) appeared to be indeterminately large when examining posterior parameter estimation distributions. This result indicates that the IMa-model, which is a very robust means of simultaneously assessing the effects of both migration and population growth on patterns in population genetics data [64], may have some difficulty fitting to this data or, alternatively, the data here may not be informative enough to discriminate peaks of such a large value on the likelihood landscape for parameter estimation. Thus, while estimates and patterns in parameters were comparable among runs and swapping rates among chains seemed sufficiently high, the lack of convergence in this one parameter limits our confidence in this analysis’ accuracy of its estimation. However, the overall pattern is consistent with results from both MIGRATE runs and mismatch distributions, as net migration favors unbalanced high rates of migration from CB to WFS (Nm _CB to WFS_ = > 2000; Nm _WFS to CB_ = 1.5) and estimates of time of divergence are within the confidence intervals of those estimated from mismatch distributions (see Table 1 for mismatch distribution data).

## Discussion

### Shelf-to-shelf connectivity in Gag

Overall, a pattern of high, asymmetric connectivity across the Gulf of Mexico was supported by multiple lines of evidence, demonstrating Campeche Bank as a genetic source for a mixed population of Gag on the West Florida Shelf. Significant, yet small φ’_ST_-values among populations, matches well with the hypothesis of on-going exchange of a small number of migrants per generation (i.e., as little as 1 Nm/generation [65]) that would lead to little genetic differentiation among populations and support for a hypothesis of on-going migration. Estimates from migration models also provided evidence for this idea, showing migrant levels > ~20 Nm per generation from CB to WFS (S4 Table). Patterns of migration were congruent with the *a priori* hypothesis of high connectivity among WFS populations and the unidirectional migration via larval dispersal from CB given the significant barriers (deep water and distance) to adult migration.

Estimates of time since expansion also suggest that asymmetric, on-going migration accounts for the continued low differentiation between populations. The timing (~100,000 ya) of population divergence of Gag populations predates the most recent glacial maximum (~18,000 ya) when sea-levels were 130 m lower than present day [66]. This climatic change would have likely led to drastic effects on population size because of shifts in available habitat and coincides with interglacial periods in the Pleistocene defined by an actively changing climate and incidences of rapid sea-level rise [67–69]. Of course, uncertainty in mutation rate estimation affects our ability to fully establish this timing. However, simulations of two populations diverging from an ancestral population with no on-going migration for 14,000 generations (calculated from time-since-divergence estimates) predicted large F_ST_-values (~0.5), which are not presented in the observed data. Additionally, simulation of the same process with the number of generation approximating time since last glacial maxima (~1300 generations) also show larger F_ST_-values than observed here (average, unstandardized F_ST_ for 100 iterations = 0.06). Thus, this “historical” population dynamic would appear to be dampened by some other homogenizing force (i.e. migration among sub-populations) and the CB population may have served as a refuge source for migrants during population expansion after range retractions.

Given the distances among populations, the biological proclivities of the species, and the apparent barriers to migration, larval dispersal would appear to be the most parsimonious explanation for persistent connectivity among sub-populations. Adult dispersal from CB to WFS is highly unlikely given that this demersal fish, typically associated with depths of 10–100 m, would have to cross a body of water over 2800 m deep. The only plausible route for adult migration between CB to the WFS would be to circumnavigate the entire Gulf, remaining on the shelf edge throughout the migration. While there is evidence for similar migrations from the Western Atlantic to the northeastern Gulf [34], no such data exist for the CB to WFS direct crossing.

With regard to larval dispersal, Fitzhugh *et al* [36] described collecting post-larval Gag in grassbeds on the WFS with birth dates at least a month prior to the earliest spawning season of WFS Gag, but consistent with spawning of Gag on CB. Physical oceanographic models indicate that shelf-to-shelf transport of CB larvae is possible via the Loop Current, the dominant current in the Gulf of Mexico, over relevant spawning months (December and January) and relevant time scales of larval dispersal (~40 days) (Dr. Steve Morey, Florida State University, personal communication). Although other studies provide supporting biological (timing of larval settlement [70]) and oceanographic (assessments of current patterns [71, 72]) evidence of the overall patterns of connectivity identified in this paper, the data are insufficient for estimating demographically meaningful effective migration rates. Regardless of the mechanism, however, this study provides one of the first examples for direct population connectivity between CB and WFS in marine fishes.

### *The regional* scale of phylogeography and ecosystems and the management and conservation of Gag

Phylogeography within the southeastern United States environments typically has focused on patterns of vicariance across the Florida Peninsula; however, this genetic “barrier” has proved largely ineffective in differentiating populations for a suite of regional marine species, leaving the actual spatial scale of many populations sometimes underappreciated. These regional patterns of genetic homogenization in other co-occurring reef fish such as red snapper and red grouper are attributed to either population expansion and retraction events [13, 32] or to persistent connectivity through migration [11, 73]. Curiously, studies in this region that have thoroughly examined the role of directionality in migration patterns are lacking (but see [74–78]). Our work with Gag suggests that unsampled and/or distant populations have a significant role in the population genetics of many of these species. Thus, the overall perception of the marine phylogeography in this region may be limited in scope.

Many studies in this region are a reflection of their spatial scale. A study by Lee and Foighil [77] that geographically overlaps our region of interest demonstrated how increasing the scale and detail of examination can lead to a greater understanding of regional phylogeography. In their study, the deep divergences observed in the scorched mussel, *Brachidontes exustus*, across the Caribbean [77] provided compelling examples where the patterns of pseudocongruence [79] in phylogeography could mislead biogeographers in their descriptions of the role of historical processes on contemporary population genetics. In fact, this mistake may be underappreciated in the literature on this region [8]. While more work on the importance of migration relative to other processes affecting population genetics and dynamics is certainly warranted, it is difficult to ignore that “ghost” populations may have larger effects on populations and wide-ranging species such as Gag and the ecosystems in southeastern maritime United States, particularly the GOM, may function at a broader scale than generally appreciated. These considerations are important in our conceptions of the evolution of species in the region, the ecological and evolutionary trajectories of their populations, the regional connectivity of subpopulations and, thus, any species specific conservation goals and/or needs.

With Gag, ignorance of the populations on CB misleads our ideas on both historical and contemporary population genetic patterns. These considerations are not only important for our understanding of regional phylogeography, but for conservation and management. Gag is one of the most sought after fisheries species in the southeastern United States. In addition to dramatic environmental incidences, Gag face many anthropogenic stresses. For example, as a result of intensive fishing pressure, its demography has been seriously altered (sex ratios have shifted from 1:6, male:female, to ~1:30 [26, 80]) and it is has been listed as experiencing overfishing on its last two stock assessments [81, 82]. Such extreme fisheries-induced selection and rapid changes in demography could likely alter patterns of genetic variation [83, 84]. Generating a more thorough understanding of the spatial processes underlying population dynamics and genetics are, thus, important to its future management and that of other species facing similar pressures as Gag. Migration studies on both ecological [34] and evolutionary [12] time scales have demonstrated that currently delineated management units from the GOM and the South Atlantic Bight are likely not biologically distinct. Given the high genetic similarities between these entities, estimates for migration rates, and larger relative genetic diversity of CB, CB is likely a source population for North Atlantic Gag within the GOM on historical and contemporary time scales and, overall, Gag populations seem connected at larger geographic scales than previously appreciated. While management units are established based on variety of political and social factors related to resource users, the role of broader regional processes may necessitate greater consideration in accurately predicting biological expectations for Gag.

Wide-spread marine species present difficult situations for ecologists and evolutionary biologists trying to explain patterns of migration given their often large effective population size, their natural variability in recruitment process, and their sometimes perturbed population status from human exploitation. Numerous studies have focused on describing patterns of differentiation in these species across a variety of spatial scales [14, 76, 85] with the intent of identifying population structure. Typically, there is often little of it or it is difficult to see (but see [86]). What remains underappreciated is explicitly examining hypotheses about why this is the case, particularly with regard to evolutionary forces affecting species on historical and contemporary time scales. Efforts have begun to tease apart the relative influence of both past and present evolutionary influences on organisms in a variety of contexts: pelagic environments [23], deep sea habitats [87], coastal areas [88], and both tropical [89] and non-tropical reefs [90]. In many, but not all, the role of larval dispersal is often paramount in explaining the patterns of genetic variation observed in these wide-ranging species. As observed in this study, the potential for long-distance dispersal during this early life history stage may result in some unexpected connections between populations.

## Supporting Information

S1 Fig Diagram of mtDNA Control Region sequence. White region indicates typical sequence, while shaded areas represent indel regions that adhere to a step-wise mutational model. Grey highlights where there is a 9-base repeat indel; black highlights where there is a 40-base repeat indel. Primers were nested within neighboring coding genes for t-RNA protein (thin black lines at the end of the sequence).
(PDF)

S1 File Gag Gulf of Mexico microsatellite data GENEPOP infile.txt: microsatellite genotypes for all samples used in this study in Genepop format. Pop1: South West Florida Shelf; Pop2: North West Florida Shelf; Pop3: Campeche Bank
(TXT)

S2 File Gag Gulf of Mexico mtDNA Control Region.fasta: sequence file of all mtDNA Control Region haplotypes used in the study; all haploptype labelled for geographic region of origin (FASTA)

S1 Table Microsatellite locus information for each region of Gulf of Mexico Gag. A_n_ is allele richness of locus; F_IS_ is inbreeding coefficient for sample; H_e_ is heterozygosity; n is the total number of alleles per locus. Symbols denote significant deviations from Hardy-Weinberg equilibrium (◊); heterozygote excess (*), and heterozygote deficiency (†).
(XLSX)

S2 Table Summary statistics on all mtDNA aggregate data, sequence only data, and indel only data of Gulf of Mexico Gag. N is sample size. L is length of sequence. h is number of haplotypes. H is haplotype diversity. S(all) is number of segregating sites. n is number of substitutions. Θ is Watterson’s (1975) estimate of the population parameter theta (= μN_e_). π is nucleotide diversity for All and Sequence Only data and average gene diversity for Indels. Significance for Tajima’s D tests and Fu’s Fs indicated by asterisks: * is p-value < 0.05; ** is p-value < 0.01; *** is p-value < 0.001.
(XLSX)

S3 Table Number of times a locus exhibited significant (uncorrected p-value < 0.05) linkage disequilibrium (LD) with another locus in each population. A less conservative Bonferroni correction of the p-value significance threshold (where α is corrected for the number of test done for each locus only, i.e. 9 pairwise comparisons, and is equal to 0.0056) showed only two significant incidences of LD in the North West Florida Shelf population, one significant incident of LD between loci in the South West Florida Shelf population, and 4 significant incidences of LD between loci on Campeche Bank. After a Bonferroni correction considering all comparisons, no specific loci pair demonstrated significant LD in multiple populations.
(XLSX)

S4 Table Estimate values of θ (= 4N_e_μ) and number of effective migrants (Nm) for different migration models from MIGRATE analysis. Regions are abbreviated as follows: Campeche Bank is CB; West Florida Shelf is WFS; South West Florida Shelf is SWFS; North West Florida Shelf is NWFS. Parameter estimates shown for each model indicate the different type of connections allowed between different populations. For example, the model describing Asymmetric Migration CB to WFS show only migration from CB to WFS allowed in this migration model. Mode, median, and 97.5% credible intervals shown for all parameters. Shade region of table indicates the model that was shown to be most supported by the data after Bayes Factor analyses (>>150—very strong support).
(XLSX)
