# Supplementary material for: From Shelf to Shelf: Assessing Historical and Contemporary Genetic Differentiation and Connectivity across the Gulf of Mexico in Gag, Mycteroperca microlepis
Source: PLoS One. 2015 Apr 9;10(4):e0120676. doi: 10.1371/journal.pone.0120676 (PMC4391813; doi:10.1371/journal.pone.0120676)
Supplement: S1 Fig — White region indicates typical sequence, while shaded areas represent indel regions that adhere to a step-wise mutational model. Grey highlights where there is a 9-base repeat indel; black highlights where there is a 40-base repeat indel. Primers were nested within neighboring coding genes for t-RNA protein (thin black lines at the end of the sequence). (PDF) [file pone.0120676.s001.pdf]

Mycteroperca microlepis mtDNA Control Region

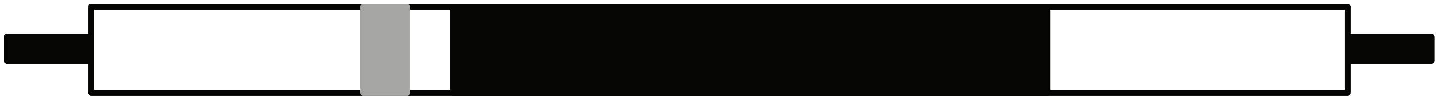

S1 Figure. Diagram of mtDNA Control Region sequence. White region indicates typical sequence, while shaded areas represent indel regions that adhere to a step-wise mutational model. Grey highlights where there is a 9-base repeat indel; black highlights where there is a 40-base repeat indel. Primers were nested within neighboring coding genes for t-RNA protein (thin black lines at the end of the sequence).
